# Associations between Blood Pressure Control and Documented Nutrition Care Using Structured Data from Electronic Health Records of Patients with Hypertension

**DOI:** 10.21203/rs.3.rs-2191063/v1

**Published:** 2023-04-11

**Authors:** April Williams, Erin L Britton, Maria D. Thomson

**Affiliations:** Virginia Commonwealth University Health System; Virginia Commonwealth University School of Medicine; Virginia Commonwealth University School of Medicine

**Keywords:** Chronic Disease, Hypertension, Preventive Care, Electronic Health Records, Nutrition

## Abstract

**Background:**

Documentation in Electronic Health Records (EHRs) of nutrition care events (overweight or obesity (BMI > 25 or 30, respectively) diagnoses, preventive care visits, or provision of patient education materials (PEM)) for chronic diseases is unclear.

**Methods:**

Cross-sectional analysis using structured EHR data from primary care visits at a health system in the US from January 2018 - December 2020 of adult patients with hypertension (n = 6,419) tested for associations between last visit blood pressure (BP) control (≤ 140 Systolic BP and ≤ 90 Diastolic BP) and aggregate nutrition care events. Descriptive statistics and multiple logistic regression models were constructed to examine the predictive power of nutrition care events for blood pressure control.

**Results:**

The median age was 62 years, 32% were male, 48% were Black, 26% were from rural areas and 35.9% had controlled BP at last visit. For the 62% of patients with documented nutrition care, 14.6% had an overweight/obesity diagnosis, 26.2% had a preventive care visit, and 42% received PEM with dietary and hypertension content. The models showed patients who had more preventive care visits (aOR 1.12; CL 1.06, 1.18) had higher odds for BP control. Whereas Black patients compared with white patients (aOR 0.84; CL 0.74, 0.95), those with more hypertension medications (aOR 0.97; CL 0.96, 0.99) and more primary care visits over the study period (aOR 0.98; CL 0.97, 0.99) had lower odds for BP control.

**Conclusions:**

In this study, documented nutrition care in preventive care visits is significantly associated with BP control, but documentation is infrequent. Additional research should include examining clinical notes for evidence of nutrition care, which may uncover areas that show promise for improving nutrition care for patients with chronic disease.

## Background

High blood pressure, or hypertension, affects nearly 1 in 3 US adults,^1^ with approximately half of cases under poor control, increasing the risk of death from a sudden heart attack or stroke.^2^ A variety of nutrition care events that occur in primary care settings may have a positive effect on patients’ dietary patterns.^3^ These could be in the form of the awareness brought to the patient through an obesity or overweight diagnosis,^4^ providers or staff spending time counseling a patient about the importance of diet to prevent, treat, or manage a chronic disease,^5,6^ or passive education materials (PEMs) containing nutrition information relevant to chronic disease management provided in an after-visit summary.^7^

Providing nutrition care may improve patient self-management and hypertension outcomes overall,^8,9^ but many primary care clinicians and staff face substantial time barriers to conducting and documenting these activities in electronic health records.^10,11^ Rates of nutrition care events are generally low,^5,12^ and their documentation in structured EHR data is largely unknown.^13,14^ A lack of documentation in the EHR may result in limited communication within the care team about patients’ nutrition statuses and challenges in evaluating care quality, which may be a missed opportunity to improve patient outcomes.^15^

Little is known about the rates of nutrition care events documented for hypertension management or their relationship with blood pressure control of patients. Given this, the objectives of this study were to assess of rates of nutrition care events identified in structured EHR data and to test whether these were associated with blood pressure control for adult patients in an academic health system. The hypothesis tested was that documented nutrition care, controlling for patient demographic and health characteristics, is associated with blood pressure control.

## Methods

### Study Design and Setting.

A single, safety-net academic medical center with six primary care clinic sites in the Mid-Atlantic US serves a diverse patient population of both rural and urban, and underrepresented racial/ethnic patients. Deidentified EHR data were obtained for clinical visits (n = 66,875) between December 2017 and December 2020 for (n = 6,419) adult patients, age 18 to 85 years, who had at least two primary care visits during the study period and had a previous diagnosis of hypertension. Records of patients with chronic kidney disease, pregnancy, or who were in hospice or long-term care were excluded per the clinical quality measure for controlling high blood pressure.^16^ Due to interruptions in clinical care resulting from the COVID-19 pandemic,^17^ records of patients who had not had at least one visit prior to March 2020 were also excluded. Patient data included demographics: sex, racial/ethnicity categories, rural geography indicated by Rural Urban Continuum codes 4–9,^18^ and insured status; and clinical factors relevant to hypertension: BMI, comorbidities,^19^ hypertension medications,^20^ number of visits, and blood pressure control.^16^ Nutrition care events were indicated by three commonly provided services in primary care hypertension management that were used as a proxy for nutrition-related information exchanged with the patient. These nutrition care events were identified by diagnosis and billing codes for overweight/obesity diagnoses and preventive visits, and a flag that diet-related education materials were provided to the patient. Clinical guidelines for the treatment of overweight/obesity include dietary behavior management,^21,22^ and the diagnosis itself may inherently offer an opportunity as a “teachable moment” behavior change intervention.^4^ Preventive visits that include evaluation for and management of chronic diseases like hypertension were chosen for the comprehensive counseling and guidance provided to patients to reduce risk factors that include diet.^23,24^ Providing patients with printed education materials is a common and passive method for counseling patients about dietary recommendations.

#### Analyses

Descriptive statistics (frequencies, proportions, means, SDs, medians, and ranges) were calculated for all patients’ and stratified samples of Black and white patients’ demographic characteristics and hypertension-relevant indicators, proportions of patients with controlled blood pressure, and rates of documented nutrition care events.

Three multiple logistic regression models were created with the outcome of patients’ blood pressure control (yes/no) at their last visit and the predictors as the total number documented in the EHR over the study period of: A) any nutrition care event, B) overweight or obesity diagnoses; preventive care visits that involve nutrition or dietary counseling; and provision of patient education materials, and C) model B stratified among data for Black patients and white patients. The models adjusted for covariates that included patient demographic characteristics age, sex, race/ethnicity (Model A only), insured status (Private/Medicaid/Medicare/None-Other), geography (rural/urban); and clinical factors BMI, number of hypertension medications prescribed during the study period, and comorbidities. The models also controlled for the random effects from a dose-response through the number of primary care visits the patient made over the study period, and geography through the inclusion of the clinic site of their last visit. Given the sample distribution and consistent disparities identified in Models A and B, Model C was constructed as a way to separately examine^25^ the strength of association between controlled blood pressure and nutrition care events among Black patient and white patient groups.

Results are presented as adjusted odds ratios and 95% confidence intervals for each multiple logistic regression model and were considered significant for *p* < 0.05. Comorbidities were calculated with HCUP Elixhauser software^19^ program using SAS Enterprise Edition 3.7 (SAS Institute, Cary, North Carolina, USA). All remaining analyses were conducted using R Statistical Software (Version 4.0.3; R Foundation for Statistical Computing, 2020). This study was approved as exempt by an Institutional Review Board.

## Results

Descriptive statistics for patients’ characteristics and clinical factors are found in Table 1. The overall median age of patients in the EHR data sample (n=6,419) was 62 years (range:18,85), nearly a third (n=2,064; 32.2%) were male, almost half (n=3,076; 47.9%) were Black/African American, 26% (n=1,668) were from rural areas, and 10.1% (n=650) were covered by Medicaid insurance. Most patients in the sample were overweight or obese (n=5,629; 87.7%) for at least one recorded BMI during the study period. The median total number of anti-hypertensive prescriptions per patient over the study period was 4 (range:0,40). About a third (n=2,275; 35.4%) had one or more comorbidities, the median number of months since hypertension diagnosis was 38 (range: 0, 229), and about a third (n=2,307; 36%) had controlled hypertension based on blood pressure recordings from their last primary care visit.

Table 2 shows a summary of documented nutrition care event rates among all the patients in the sample, as well as rates stratified by Black and white patient groups. Across clinics, the median number of nutrition care events documented in the EHR data was 1 (range:0,16) per patient during the study period. Many patients (n=3,983; 62.1 %) had at least one documented nutrition care event over the study period, ranging from 61.9% (white patients; n=1,819) to 63.3% (Black patients; n=1,948). Distribution of PEMs was the most frequent nutrition care event documented for patients (n=2,698; 42%), ranging from 35.9% (Black patients; n=1,330) to 42.1% (white patients; n=1,239). Overweight/obesity diagnoses were recorded for far fewer patients overall (n=938; 14.6%), ranging from 11.1% (white patients; n=327) to 18.3% (Black patients; n=563). Preventive visits were documented for a little over a quarter of total patients (n=1,683; 26.2%) at rates ranging from 24.9% (Black patients; n=765) to 27.5% (white patients; n=807).

### Blood Pressure Control and Nutrition Care Events

The multiple logistic regression model (Model A) that tested for association between blood pressure control and the total number of documented nutrition care events (AUC: 0.585) found no significant association (aOR: 1.03; 95% CI: 1.00, 1.06). However, the model (Model B) that tested for associations between blood pressure control and the number of specific types of nutrition care events (AUC: 0.590) had some significant findings. Associations with demographic and health covariates were consistent for each model, with race/ethnicity and the number of hypertension medications prescribed over the study period significantly associated with blood pressure control. Patients with more preventive care visits and more hypertension medications documented across the study period had higher odds for having controlled blood pressure (aOR 1.12; 95% CI: 1.06, 1.18 and aOR 0.97; 95% CI: 0.96, 0.99, respectively) after adjusting for age, sex, race/ethnicity, insurance, geography, BMI, comorbidity index, and clinic site for their last visit. Results of Model B produced similar results regarding preventive care visits associated with higher odds for controlled blood pressure among Black patients (aOR 1.12; 95% CI: 1.04, 1.21) and white patients (aOR 1.11; 95% CI: 1.03, 1.20). Full model results are presented in Table 3.

### Patient Demographics and Blood Pressure Control

Non-Hispanic Black patients, compared with non-Hispanic white patients had consistently lower odds for controlled blood pressure in both logistic regression models A and B (aOR: 0.82-0.84; 95% CI: 0.73-0.74, 0.93-0.95), but Hispanic ethnicity and all other race/ethnicities did not have significantly different odds for controlled blood pressure compared with the non-Hispanic white patients in the sample. Among white patients, those who were covered by Medicaid insurance had lower odds for controlled blood pressure (aOR: 0.61; 95% CI: 0.41, 0.90). No additional significant differences were detected for controlled blood pressure across the other demographic factors examined in these models.

### Clinical Factors and Blood Pressure Control

In both Models A, B, and C among Black patients, the more hypertension medications prescribed over the study period (aOR: 0.96-0.97; 95% CI: 0.95-0.97, 0.99) and more visits (aOR: 0.98; 95% CI: 0.98, 0.99) had lower odds for controlled blood pressure. However, there were no significant differences detected for BMI category or number of comorbidities.

## Discussion

EHR data was used in this study to examine associations between blood pressure control and nutrition care events identified through documentation of clinical activities that imply nutrition or diet information was communicated with patients who have hypertension. Visits reported in the EHR data were at primary care clinics part of a health system that serves a diverse population of rural and underrepresented racial/ethnic patients. Among the 6,419 patients with hypertension, rates of nutrition care events were generally low, although documentation was variable across the clinics. Of documented nutrition care events, preventive care visits where counseling about dietary risk factors occured were found to be associated with improved odds for blood pressure control. However, there were disparities in blood pressure control by race, for those who had more prescribed hypertension medications, and those who had more clinical encounters.

Overall low rates of documented nutrition care events found in this study support the wider call to address barriers faced by clinicians and staff such as time pressures and limited provider education in providing nutrition care in primary care settings.^10,11^ To understand how these rates might increase, these clinical activities must be measured, and to be measured, they must be documented. Stange and colleagues argued health systems measure what is valued,^26^ and this study highlights a few important opportunities where nutrition is addressed with patients: diagnoses of overweight or obesity diagnoses, preventive care visits, and provision of patient education materials.

In this study, an overweight or obesity diagnosis was not associated with controlled blood pressure However, BMI persists as a clinical tool for risk assessment, and the diagnosis, as previously mentioned, could play an important role in patient awareness of a health risk. Mixed findings about BMI associations with hypertension management is seen in a recent study of blood pressure control rates among adults with hypertension. Foti and colleagues found those with overweight or obesity had higher rates of blood pressure control compared with those who had lower BMI. This, the authors suggested, might be because the lower BMI patients may be less aware of their hypertension and be treated less often or intensely.^27^ Interestingly, rates of obesity and overweight diagnoses were not concordant with BMI calculated from patient chart data in the present study. The disconnect between BMI and documented obesity diagnoses has been shown elsewhere; one study of EHR data found that while more than half (52%) of patients in the sample had a BMI ≥ 30.0 qualifying them for an obesity diagnosis, very few (5.6%) had obesity recorded in their health record problem list.^28^ One factor contributing to the lack of documenting or addressing overweight or obesity with patients may be clinicians are ill-prepared to discuss the topic.^29^

The present study had more than a quarter of patients who had at least one preventive visit documented in their EHR record that included a discussion of nutrition-related risk factors. Clinical guidelines suggest nutrition counseling is included in lifestyle treatment for chronic diseases for which diet is a risk factor. Healthy People 2020 suggested a goal to *“increase the proportion of physician office visits made by patients with a diagnosis of cardiovascular disease, diabetes, or hyperlipidemia that include counseling or education related to diet or nutrition,”*^30^ from 11.5–12.7% (a 10% increase) by 2020 as measured by the National Ambulatory Medical Care Survey (NAMCS). Overall rates reached over 20% by 2015, which may be why the objective was eliminated altogether for Healthy People 2030. However, rates assessed by the present study and Healthy People remain objectively low for a service that may be universally useful if it were achievable to provide such service to all patients, as preventive visits have long been shown to help improve patient health outcomes.

Of note, preventive care visits were less common for Black patients compared to white patients. Given blood pressure control was less likely among Black patients, and in overall and stratified analysis for both Black patient and white patient samples, preventive care visits were positively and significantly associated with blood pressure control. This suggests a disparity in service uptake that may be a missed opportunity for which further understanding and improvements are needed. Others have researched and found delays and underutilization of health services by Black patients due to a variety of reasons^31^ including socioeconomic barriers,^32^ medical mistrust^33^ and perceived racism.^34^

Leveraging data entered into the patient’s EHR is a way to support the care team in prioritizing appropriate preventive care depending upon patients’ individual needs. Krist, et. al. developed an EHR intervention that incorporated automated, tailored, patient-centered messaging for preventive care. In a summary of the initial six months of its use (November 2010 through May 2011), clinics were successful in using the tool to support clinicians to counsel patients about health behaviors and customize prevention and treatment plans for patients.^35^ Such initiatives have been instituted within various health systems and clinics nationally over the past decade to support clinicians through the use of risk calculators,^36^ automated prompts for clinicians and patients,^37^ and provision of printed or electronic patient education materials.^38^ Although patient education materials were not significantly associated with blood pressure control in this study, they were the most commonly provided nutrition care event. Timely research is needed to understand the effects of using these tools in primary care and preventive visits on patient outcomes across different populations in order to identify disparities as have been identified in this study, and apply their use in appropriate settings and support guidelines to do such. Due to the fast rate at which technology use in health care has advanced, health services researchers face additional challenges in efficient dissemination and evaluation of EHR process implementations.^39^

Findings of this study offer new contributions and suggestions for additional work needed within the burgeoning area of health services research that seeks to improve preventive services to support patients with chronic disease. First, due to our limited established structures to measure or examine nutrition-related activities in primary care, this study provides a framework of proxy measures for preventive nutrition care that may be found in the voluminous EHR data. Next, this study applied analyses in stratified samples of underrepresented racial and ethnic patients, with significant findings to support conducting further detailed explorations of causes and ways forward for improvement. Although not part of the present study, additional stratification by comorbidities may be conducted to assess differences in treatment plans and nutrition care for complex patients. To gain more insight into nutrition care services and relevant patient outcomes, future research should use a larger data sample or data from a group of health systems, and additional data sources e.g., clinical notes for preventive counseling; and claims data for clinical referrals to dietitians are other avenues to offer a more robust picture of successful processes for conducting and documenting nutrition care delivery that might reveal targets for improvements.

Our study has important limitations. Using EHR data for research has known challenges due to variability of data entry.^40^ As a single healthy system study, findings may not be generalizable, although the EHR used is widely used, and clinics part of the health system served diverse patient populations that represent higher risk for health disparities. These services are critical to chronic disease management and challenges related to their documentation and delivery to patients remain globally applicable.

## Conclusions

The present study utilized EHR data to provide a description of nutrition care delivery and examination of its association with patient blood pressure outcomes within a single health system. Overall, documentation of received nutrition care events was low, and preventive care visits, but not overweight/obesity diagnosis or delivery of patient education materials, was associated with patients’ blood pressure control. Additionally, disparities were identified in rates of nutrition care events and odds for blood pressure control by race. Further research is needed to explore ways to improve documentation and equity of nutrition care. Enhancing EHR workflows, education for providers and staff about nutrition care, and improving effective communication and collaboration within the clinical team may all be system-level targets for improving nutrition care and hypertension outcomes.

## Figures and Tables

**Table 1:** Patient Demographics and Hypertension Risk Factors

	Total Patients^a^ (n=6,419)	Black Patients (n=3,076)	White Patients (n=2,937)
**Median Age (range)**	62 (18,85)		
**Male n (%)**	2064 (32.2)	787 (25.6)	1112 (37.9)
**Race/Ethnicity n (%)**			
Non-Hispanic Black	3076 (48.0)	-	-
Non-Hispanic white	2937 (45.8)	-	-
Hispanic	99 (1.5)	-	-
Other Race/Ethnicity^b^	307 (4.8)	-	-
**Rural^c^ n (%)**	1668 (26)	534 (17.4)	1115 (38)
**Medicaid n (%)**	650 (10.1)	448 (14.6)	155 (5.3)
**Mean BMI^d^ (SD)**	34.9 (14.3)	36.6 (15.8)	33.8 (13.1)
**Current Smoker^e^ n (%)**	488 (7.6)	299 (9.7)	181 (6.2)
**Current Alcohol^f^ Use n (%)**	581 (9.1)	376 (12.2)	181 (6.2)
**Median Hypertension Medications^g^ (range)**	5 (0,40)	4 (0,40)	4 (0,24)
**One or More Comorbidities^h^ n (%)**	2275 (35.4)	1211 (39.4)	889 (30.3)
**Median Months Since Hypertension Diagnosis (range)**	38 (0,229)	48 (0,229)	32 (0,224)
**Controlled Blood Pressure^i^ n (%)**	2307 (36)	1012 (32.9)	1149 (39.1)

Number of patients based on patient’s last visit.

Other Race/Ethnicity includes Asian, American Indian-Alaskan, Native Hawaii/Other Pacific Islander, Other, or unknown race/ethnicities.

Patients residing in rural areas was determined using patient’s zip code corresponding to RUCC code (4-9). Missing n = 258 (4.0%); missing from Black patients: 67 (2.2%); missing from white patients: 180 (6.1%).

BMI is calculated as the maximum recorded BMI across the study period per patient If BMI was not available, the patient’s height and maximum weight across the study period were used to calculate BMI using the standard formula (weight (kg) / height (cm) / height (cm)] x 10,000). Missing n = 70 (1.1%); missing from Black patients: 43 (1.4%); missing from white patients: 24 (0.8%).

Smoking status at last visit missing n = 4435 (64.9%); missing from Black patients: 1860 (60.5%); missing from white patients: 1876 (63.87)

Alcohol status at last visit missing n = 5488 (82%) missing from Black patients: 2186 (73%); missing from white patients: 2575 (90)

Hypertension medications were the total prescribed across the study period per patient. Medications were identified using the HEDIS reference list for hypertension medications^20^ and reviewed with two physicians boarded in internal medicine prior to making calculations.

Comorbidities calculated as mode of Elixhauser Comorbidity Index across the study period per patient. The index was calculated using the Healthcare Cost and Utilization Project Methods Series Comorbidity Software. The comorbidity index is a total sum of diagnosed chronic disease comorbidities found in 31 categories defined in the software.^21^

Patients with controlled hypertension (≤140 mm Hg SBP and ≤90 mm Hg DBP) per the National Committee for Quality Assurance (NCQA) quality measure for “Controlling High Blood Pressure”^22^ were flagged (yes/no) and aggregated for clinic-level patient panel proportions. Missing SBP and DBP at the last visit was counted as uncontrolled.

**Table 2:** Rates of Nutrition Care Events

	Total Patients (n=6,419)	Black Patients (n=3,076)	White Patients (n=2,937)
**Median Nutrition Care^a^ (range)**	1 (0, 16)	1 (0, 16)	1 (0, 15)
**Nutrition Care^b^ n (%)**	3983 (62.1)	1948 (63.3)	1819 (61.9)
**Patient Education Materials^c^ n (%)**	2698 (42.0)	1330 (35.9)	1239 (42.1)
**Overweight/Obesity Diagnosis^d^ n (%)**	938 (14.6)	563 (18.3)	327 (11.1)
**Preventive Visits^e^ n (%)**	1683 (26.2)	765 (24.9)	807 (27.5)

Number of nutrition care services documented per patient across the study period. Services were flagged (yes/no) based on the list of International Classification of Diseases version 10 (ICD-10) diagnosis and Current Procedural Terminology (CPT) codes.

Number of patients who received at least one form of nutrition care across the study period.

Number of patients who received at least one patient education material (PEM) across the study period.

Number of patients who were diagnosed with overweight or obesity (ICD-10: Z68.XX; Z71.3; R63.5; E66.XX; E88.81) across the study period.

Number of patients who were provided with a preventive care visit (CPT: 99381-99387, 99391-99397) that includes counseling about chronic disease risk factors and management strategies across the study period.

**Table 3.** Adjusted Odds Ratios for Blood Pressure Control^a^

	Blood Pressure Control OR (95% CI)			
	Model A^b^ (n=6,076)	Model B^c^ (n=6,076)	Model C among Black Patients^d^ (n=2,957)	Model C among white Patients^e^ (n=2,727)
**Total Number Nutrition Care Events**	1.03 (1.00, 1.06)			
**Overweight/Obesity Diagnoses**		1.04 (0.98, 1.11)	1.05 (0.96, 1.14)	1.06 (0.95, 1.18)
**Preventive Care Visits**		**1.12 (1.06, 1.18)**	**1.12 (1.04, 1.21)**	**1.11 (1.03, 1.20)**
**Patient Education Materials**		0.97 (0.93, 1.02)	0.96 (0.90, 1.02)	0.98 (0.92, 1.04)
**Age^f^**	1.00 (1.00, 1.00)	1.00 (1.00, 1.00)	1.00 (1.00, 1.00)	1.00 (1.00, 1.00)
**Sex**				
Male	1.01 (0.89, 1.14)	1.01 (0.89, 1.14)	0.87 (0.72, 1.05)	1.23 (1.03, 1.20)
**Race/Ethnicity**				
Non-Hispanic Black	**0.82 (0.73, 0.93)**	**0.84 (0.74, 0.95)**	-	-
Hispanic	0.83 (0.53, 1.28)	0.82 (0.52, 1.26)		
Other Race/Ethnicities	0.97 (0.74, 1.25)	0.98 (0.76, 1.27)		
**Insurance**				
Medicaid	0.90 (0.75, 1.09)	0.93 (0.77, 1.13)	1.12 (0.89, 1.42)	**0.61 (0.41, 0.90)**
Medicare	0.95 (0.82, 1.11)	1.02 (0.87, 1.18)		
None/Other	1.01 (0.67, 1.50)	1.05 (0.70, 1.56)		
**Geography**				
Rural	1.00 (0.75, 1.33)	1.01 (0.75, 1.34)	1.07 (0.70, 1.61)	0.87 (0.57, 1.32)
**BMI Category**				
25-30 (Overweight)	1.01 (0.83, 1.22)	1.01 (0.83, 1.22)	1.08 (0.78, 1.49)	0.91 (0.70, 1.18)
>30 (Obese)	0.94 (0.78, 1.12)	0.94 (0.74, 1.12)	1.04 (0.77, 1.40)	0.79 (0.62, 1.01)
**# Medications prescribed during study period**	**0.97 (0.96, 0.99)**	**0.97 (0.96, 0.99)**	**0.97 (0.95, 0.99)**	0.98 (0.96, 1.01)
**Comorbidity Index**	0.99 (0.91, 1.08)	0.98 (0.91, 1.08)	0.97 (0.86, 1.10)	1.03 (0.90, 1.17)
**Number of Visits**	**0.98 (0.97, 0.99)**	**0.98 (0.97, 0.99)**	**0.98 (0.97, 0.99)**	0.99 (0.97, 1.00)

**Bold** odds ratios are significant (*p*<0.05).

Multiple Logistic Model A (AUC: 0.586): Patient blood pressure control at last visit (yes/no) ~ Total # Nutrition Care over study period + Age^2^ + Sex + Race/Ethnicity compared to reference white + Insurance compared to reference Private Insurance + Rural/Urban + BMI compared to reference BMI 18.9-24.9 + # Hypertension Medications over study period + # Months Since Hypertension Diagnosis at last visit + Comorbidity Index mode over study period + Number of Visits + Clinic (not significant, not shown)

Multiple Logistic Model B (AUC: 0.590): Patient blood pressure control at last visit (yes/no) ~ Total # Overweight-Obesity Diagnoses + Preventive Care Visits + Patient Ed Materials + Age^2^ + Sex + Race/Ethnicity compared to reference white + Insurance compared to reference Private Insurance + Rural/Urban + BMI compared to reference BMI 18.9-24.9 + # Hypertension Medications over study period + # Months Since Hypertension Diagnosis at last visit + Comorbidity Index mode over study period + Number of Visits + Clinic (not significant, not shown)

Multiple Logistic Model C among black patients(AUC: 0.596): Patient blood pressure control at last visit (yes/no) ~ Total # Overweight-Obesity Diagnoses + Preventive Care Visits + Patient Ed Materials + Age^2^ + Sex + Insurance compared to reference Private Insurance + Rural/Urban + BMI compared to reference BMI 18.9-24.9 + # Hypertension Medications over study period + # Months Since Hypertension Diagnosis at last visit + Comorbidity Index mode over study period + Number of Visits + Clinic (not significant, not shown)

Multiple Logistic Model C among white patients (AUC: 0.592): Patient blood pressure control at last visit (yes/no) ~ Total # Overweight-Obesity Diagnoses + Preventive Care Visits + Patient Ed Materials + Age^2^ + Sex + Insurance compared to reference Private Insurance + Rural/Urban + BMI compared to reference BMI 18.9-24.9 + # Hypertension Medications over study period + # Months Since Hypertension Diagnosis at last visit + Comorbidity Index mode over study period + Number of Visits + Clinic (not significant, not shown)

Age transformed to age^2^
